# Aflibercept 8 mg treat-and-extend pathway for the treatment of neovascular age-related macular degeneration: guidance from a UK expert panel

**DOI:** 10.1038/s41433-025-04180-8

**Published:** 2026-01-16

**Authors:** Richard Gale, Mahmoud Husseiny Awad, Clare Bailey, Peter Cackett, Ramandeep Chhabra, Louise Downey, Faruque Ghanchi, Ajay Kotagiri, Nirodhini Narendran, Ian Pearce, Tunde Peto, Sobha Sivaprasad, Saad Younis, Jackie Napier, Rose Gilbert, Safeen Ismat

**Affiliations:** 1 HYMS, University of York, York Hospital, York and Scarborough Teaching Hospitals NHS Foundation Trust, York, UK; 2 Singleton Hospital, Swansea Bay University Health Board, Swansea, Wales UK; 3 Bristol Eye Hospital, University Hospitals Bristol and Weston NHS Foundation Trust, Bristol, UK; 4 Princess Alexandra Eye Pavilion, NHS Lothian, Edinburgh, Scotland UK; 5 Manchester Royal Eye Hospital, Manchester University NHS Foundation Trust, Manchester, UK; 6 Hull and East Yorkshire Eye Hospital, Hull University Teaching Hospitals NHS Trust, Hull, UK; 7 Bradford Teaching Hospitals NHS Foundation Trust, Bradford, UK; 8 Sunderland Eye Infirmary, South Tyneside and Sunderland NHS Foundation Trust, Sunderland, UK; 9 The Royal Wolverhampton NHS Trust, Wolverhampton, UK; 10 School of Life and Health Sciences, Aston University, Birmingham, UK; 11 NHS University Hospitals of Liverpool Group, Liverpool, UK; 12 Queen’s University Belfast, Belfast, Northern Ireland UK; 13 NIHR Moorfields Biomedical Research Centre, Moorfields Eye Hospital NHS Foundation Trust, London, UK; 14 Western Eye Hospital, Imperial College Healthcare NHS Trust, London, UK; 15 Medical Department, Bayer plc, Reading, UK

**Keywords:** Macular degeneration, Drug therapy

## Abstract

**Background/objectives:**

Therapies with robust visual outcomes and reduced patient and healthcare system treatment burden are needed amidst the rising incidence of neovascular age-related macular degeneration (nAMD). Aflibercept 8 mg is an additional treatment option with demonstrated potential for extended dosing intervals of up to 24 weeks. The objective of this publication is to introduce a clinical care pathway, developed by expert consensus of experienced UK clinicians, to support best practice with aflibercept 8 mg in nAMD.

**Methods:**

A structured, face-to-face roundtable meeting of 13 UK retina specialists was held on 8 October 2024, organised and funded by Bayer. The expert panel reached consensus following review of key clinical trial data and consideration of current NHS clinical practice to provide guidance on the use of intravitreal aflibercept 8 mg in nAMD.

**Results:**

The panel provided recommendations for an aflibercept 8 mg treat-and-extend pathway for both treatment-naïve and previously treated patients with nAMD. Criteria were developed to guide dosing interval extension, reduction or maintenance based on visual acuity and optical coherence tomography imaging. More detailed guidance includes considerations for switching treatments to or from aflibercept 8 mg, monitoring, and discontinuing treatment.

**Conclusions:**

Aflibercept 8 mg may offer opportunities for longer treatment intervals and reduced patient and clinic burden compared with first-generation agents. The proposed treatment pathway is practical and accounts for variability in healthcare structures and capacity pressures, providing clinicians with flexibility in implementing these recommendations while addressing patient needs.

## Introduction

A 2024 report from The Royal College of Ophthalmologists (RCOphth), which evaluated 26,847 eyes from 24,300 patients starting treatment for neovascular age-related macular degeneration (nAMD), highlighted the pressures facing eye care provision in the UK. In 2021–2022, only 21% of eyes with referral data (2151 out of 10,279) received their first vascular endothelial growth factor (VEGF) antagonist (anti-VEGF) injection within the recommended 2 weeks from primary care referral, and only 66% of eyes (17,730 out of 26,847 eyes) completed their three loading injections within the recommended 10 weeks of the first treatment [1]. The report also highlighted high rates of patients without documented follow-up [1]. These issues are likely to be linked to ongoing workforce challenges in UK National Health Service (NHS) eye units, exacerbated by increasing demand for retina services and pre-existing patient backlog [2]. Meanwhile, the burden of age-related macular degeneration is rising and is projected to cause sight loss or blindness in 1.23 million people in the UK by 2050 [3].

A report from Moorfields Eye Hospital, a large tertiary hospital in the UK, predicted that its retinal care unit will need to accommodate the administration of ~83,000 intravitreal injections in 2029, a significant rise from ~45,000 in 2019 [4]. Clinics under strain face significant direct and indirect medical and non-medical health system costs [5]. As such, optimising service delivery [6] and using therapies with a reduced treatment burden (i.e. more durable therapies) are recommended to address the increase in demand for retina services [3]. Therapies that deliver robust visual outcomes while reducing the burden of treatment on patients and service providers can also be cost-effective even if their unit price is higher than that of other drugs [5].

Since the introduction of the first-generation anti-VEGF treatments and their subsequent biosimilars in the UK (pegaptanib in 2006, ranibizumab in 2007 and aflibercept 2 mg in 2012), treatment options have expanded, with several second-generation agents now available (brolucizumab in 2020, faricimab in 2022 and aflibercept 8 mg in 2024). The efficacy and safety of aflibercept 8 mg at extended dosing intervals were investigated in PULSAR (NCT04423718), a 96-week, phase III, non-inferiority trial involving 1011 treatment-naïve patients [7]. Patients were randomised 1:1:1 to receive treatment with either aflibercept 2 mg at 8-week (q8; *n* = 337) fixed intervals or aflibercept 8 mg at 12-week (q12; *n* = 336) or 16-week (q16; *n* = 338) intervals; 1009 patients (336, 335 and 338, respectively) completed the 48-week period and were included in the safety and full analysis sets. After three initial monthly doses of the respective formulation (loading phase), patients who received aflibercept 8 mg could have their treatment intervals adjusted in accordance with prespecified dose regimen modification (DRM) criteria [7], whereas intervals for patients treated with aflibercept 2 mg remained fixed at 8 weeks.

Outcomes of the trial included non-inferior best corrected visual acuity (VA) gains from baseline with aflibercept 8 mg versus aflibercept 2 mg at Week 48 (primary endpoint) and superior drying effect (resolution of macular fluid in the central subfield, defined as a 1 mm diameter circle around the fovea) following the matched loading phase at Week 16, a key secondary endpoint [7]. Of all subjects, 63% of patients (422 out of 667) treated with aflibercept 8 mg (q12 and q16 arms combined) were dry in the central subfield compared with 52% (173 out of 335) of those treated with aflibercept 2 mg at Week 16 [7]. Although interval shortening was permitted in the aflibercept 8 mg arms after the loading phase, 83% of patients (523 out of 628) randomised to aflibercept 8 mg maintained treatment intervals of ≥12 weeks until Week 48 [7]. Dosing interval extensions were allowed only after the first year of the study (Week 48 onwards). At Week 96, 87% of patients in the 8q12 group had achieved ≥12-week dosing intervals, while 78%, 53% and 31% of patients in the 8q16 group qualified for last assigned dosing intervals of ≥16 weeks, ≥20 weeks and 24 weeks, respectively, while maintaining visual and anatomic outcomes [8].

Aflibercept 8 mg has been available for the treatment of nAMD and visual impairment due to diabetic macular oedema in the UK since January 2024 [9]. The aflibercept 8 mg licensed posology is the same for both indications and consists of three initial monthly injections, after which clinicians can use their discretion to treat patients at intervals of 8–16 weeks for the fourth injection and 8–24 weeks for all subsequent injections. The shortest interval between injections while maintaining stable visual and/or anatomic outcomes is 8 weeks and the longest is 24 weeks. Table 1 highlights the licensed posology of aflibercept 8 mg and aflibercept 2 mg in nAMD, as of August 2025 [9, 10].

**Table 1:** Licensed posology of aflibercept 8 mg and 2 mg in nAMD (correct as of August 2025).

Aspect of posology	Aflibercept 8 mg [9]	Aflibercept 2 mg [10]
Loading doses	Three initial monthly injections	Three initial monthly injections
Action immediately after loading	Extend treatment interval to 8–16 weeks	Extend treatment interval to 8 weeks
Further changes to the treatment regimen during the maintenance phase	Individualise treatment, such as by using a treat-and-extend regimen	Maintain treatment at 8 weeks or individualise, such as by using a treat-and-extend regimen
Minimum interval between injections when treatment is individualised	8 weeks	4 weeks
Maximum interval between injections when treatment is individualised	24 weeks	16 weeks
Recommended duration of adjustments to injection intervals^a^	At clinician’s discretion	2 or 4 weeks

Licensed posology is often provided in months. For full, up-to-date details of the licensed posology, always refer to the relevant and current summary of product characteristics.

*nAMD* neovascular age-related macular degeneration.

^a^Applies to interval extensions. Please note the minimum and maximum intervals between injections.

A number of different posologies are used in the UK for the delivery of intravitreal anti-VEGF treatment, including fixed dosing, *pro re nata*, treat-and-extend (T&E), personalised treatment interval and DRM (or retreatment criteria) [6, 11]. NHS England, in their June 2025 document titled ‘Commissioning Guidance: Medical Retinal Treatment Pathway in Wet Age-related Macular Degeneration’ and the RCOphth both recommend implementing T&E regimens for nAMD, with treatment intervals determined according to ongoing monitoring of VA and optical coherence tomography (OCT) findings [3, 12]. T&E dosing has been found to achieve efficacy comparable to that of fixed dosing while resulting in significantly lower treatment burden and better VA outcomes relative to *pro re nata* regimens [11]. Furthermore, the RCOphth and NHS England also suggest, in consultation with the patient, to consider switching therapy to a more durable alternative anti-VEGF agent as rescue in cases of poor response, where treatment harmonisation is required, or to reduce treatment burden [3, 12]. Following pivotal phase III/IV studies such as ALTAIR (NCT02305238) [13], ARIES (NCT02581891) [14] and TENAYA/LUCERNE (NCT03823287/NCT03823300) [15], expert consensus recommendations informed by experience in clinical practice have been developed for T&E regimens with aflibercept 2 mg [16, 17] and switching to either faricimab from an alternative anti-VEGF agent [18] or aflibercept 2 mg from ranibizumab biosimilars [19].

The objective of this publication is to introduce a clinical care pathway to support best practice with aflibercept 8 mg in both treatment-naïve and previously treated patients with nAMD. It also aims to provide guidance on clinical assessment points to facilitate such pathways in UK local practice. The recommendations are designed to be practical, considering the diverse capacity and resource pressures faced by many NHS healthcare units in a real-world setting.

## Methods

A structured face-to-face roundtable meeting of 13 UK retina specialists was held on 8 October 2024, organised and funded by Bayer. The objective was to develop a consensus treatment pathway and provide guidance for the use of aflibercept 8 mg in managing patients with nAMD, considering both clinical outcomes and service efficiency.

The expert panel reviewed key clinical trial data and considered current clinical practice and the use of intravitreal aflibercept 8 mg in nAMD. The development of the pathway was facilitated by focused discussions to reach consensus, addressing treatment initiation with aflibercept 8 mg, dosing interval adjustments and switching strategies.

## Guidance from a UK expert panel

### Criteria for treatment initiation in treatment-naïve patients

Aflibercept 8 mg can potentially be used across all adult patient populations with nAMD, for all subtypes of nAMD, including those with polypoidal choroidal vasculopathy, with similar effectiveness anticipated.

The safety profile of aflibercept 8 mg is consistent with that of aflibercept 2 mg [9], with no additional risks identified for adverse events such as retinal pigment epithelial detachment, rips or intraocular inflammation [7, 9]. Similarly to all intravitreal agents, aflibercept 8 mg is contraindicated in the presence of hypersensitivity to the active substance; active ocular or periocular infection; and active, severe intraocular inflammation [9].

Data from the PULSAR trial did not identify any clinically meaningful differences in increased risk of raised intraocular pressure with aflibercept 8 mg despite the slightly larger intravitreal injection volume compared with aflibercept 2 mg (0.07 ml vs. 0.05 ml) [7]. Patients with glaucoma should, therefore, continue to be managed per local practice, informed by national recommendations [3, 20]. In the rare circumstance of administration to patients with very advanced disc cupping, consideration should be given to this additional volume. As with other anti-VEGF therapies, it is recommended not to inject patients with poorly controlled glaucoma while intraocular pressure is ≥30 mmHg.

Where possible, the aflibercept 8 mg pre-filled syringe (OcuClick™) should be used. The mechanical dose setting mechanism of the device ensures precise delivery of the aflibercept 8 mg dose and simplifies the priming procedure in comparison to that of the aflibercept 2 mg pre-filled syringe. The narrow diameter of the syringe barrel and longer plunger travel distance facilitate slow administration of the drug into the eye, reassuring clinicians about the risk of post-injection intraocular pressure spike. In addition, single sterile, pre-filled devices reduce the opportunities for infection compared with vials, which require multiple steps and components (such as a sterile needle, a syringe and drawing up from the vial), thus lowering the potential risk of endophthalmitis [21, 22].

### Overview of the aflibercept 8 mg treatment pathway

**Fig. 1:**
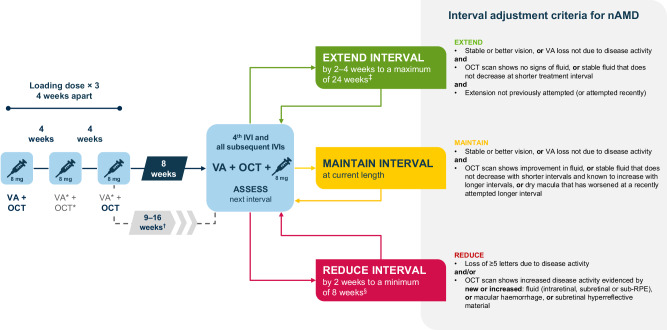
Intravitreal aflibercept 8 mg T&E pathway for the treatment of patients with nAMD. *Optional. ^†^Extending the interval between the 3rd and 4th injections beyond 8 weeks is at the discretion of the clinician and in consultation with the patient, providing the OCT at the 2nd and 3rd injection visit indicates no disease activity or that disease activity is controlled. Interim monitoring with OCT may be required. ^‡^Increase interval by 4 weeks in treatment-naïve patients and by 2 weeks in patients with previous interval reduction or those who have been switched from an alternative anti-VEGF treatment owing to suboptimal response to treatment. Treatment intervals greater than 20 weeks between injections have not been studied. In extended dosing intervals of 24 weeks, monitoring between injections may be considered. ^§^Treatment intervals of shorter than 8 weeks between injections have not been studied. If visual and anatomic outcomes indicate that the patient is not benefiting from continued treatment, aflibercept 8 mg should be discontinued. *IVI* intravitreal injection, *nAMD* neovascular age-related macular degeneration, *OCT* optical coherence tomography, *RPE* retinal pigment epithelium, *T&E* treat-and-extend, *VA* visual acuity, *VEGF* vascular endothelial growth factor.

The consensus aflibercept 8 mg T&E pathway is presented in Fig. 1. In summary, treatment initiation starts with three loading doses 4 weeks apart. The fourth injection is given 8 weeks after the third loading dose, though this interval may be extended up to 16 weeks under certain circumstances. VA assessment and OCT imaging guide adjustments to treatment intervals, which can be extended, maintained or reduced.

#### Loading phase

In line with the aflibercept 8 mg licensed posology, the loading phase consists of three consecutive injections, 1 month apart. The loading doses should be prioritised because early treatment, completion of the three loading doses and adherence to treatment plans give the best opportunity for satisfactory long-term outcomes.

It is recommended that pre-injection OCT imaging be performed at the first and third injection visits to inform subsequent treatment decision-making. OCT imaging at every injection visit can also provide useful data and is a common practice. VA assessment should be mandatory at baseline and should ideally be performed during the initiation phase as well, although this is at the discretion of the treating clinician; VA and fundoscopy may be omitted if the clinical picture and OCT are favourable during the loading phase. At all subsequent visits where a retreatment interval decision is required, OCT and VA assessments should be performed. Considering all regular intravitreal treatment may lead to changes in intraocular pressure [23], it is at the discretion of the clinician to consider baseline optic disc OCT to evaluate retinal nerve fibre layer thickness at annual intervals or more frequently as required.

#### Maintenance phase

If there has been no clinical improvement in anatomic or visual outcomes at the third visit (i.e. after two injections) the diagnosis of nAMD should be revisited and confirmed. If there is no disease activity or the disease has improved compared with baseline based on OCT findings, the fourth injection should be administered 8 weeks after the third dose. In certain cases, clinicians may consider extending the treatment interval after the three initial monthly injections up to a maximum of 16 weeks, if OCT and VA are favourable at the third visit (no disease activity). In such a case, informed discussion with the patient and consideration of interim OCT monitoring (remotely or in person) are paramount. This approach is supported by the results of the PULSAR trial, in which 79% (251 out of 316) and 77% (239 out of 312) of treatment-naïve patients randomised to either q12 or q16 aflibercept 8 mg (after a loading phase) maintained good visual and anatomic outcomes and stayed on their assigned intervals up until Week 48 [7]. In previously treated patients who switched to aflibercept 8 mg because of suboptimal response and requirement of short intervals, immediate extension to 16 weeks should not be considered.

At the fourth visit, a T&E protocol would guide injections with extension of intervals by 4 weeks; in patients who have previously demonstrated a suboptimal response to treatment with an alternative anti-VEGF agent, interval extension by 2 weeks is recommended. Subsequent treatment intervals are either extended, maintained or reduced with the potential of reaching the maximum licensed interval (24 weeks as of August 2025) [9], provided there is consideration of monitoring. Criteria for each decision are aligned with previous recommendations [16] and shown in Fig. 1. In patients who have previously required an interval reduction with aflibercept 8 mg, short interval extensions of 2 weeks are recommended. If active disease arises during the extension phase, the interval should be reduced to the last known interval that offered disease stability (minimum every 8 weeks); treating clinicians should consider fixing the interval for three further doses before any subsequent attempt to extend. In patients who exhibit new, unexpected disease activity during a long treatment interval (≥16 weeks), or who experience major disease reactivation (e.g. multiple abnormal findings on OCT, significant loss of vision, or large macular haemorrhage) at any interval, clinicians may consider reducing the next interval directly to a minimum of every 8 weeks or even re-loading patients with three doses 1 month apart. Note that monthly doses of aflibercept 8 mg have not been studied for more than three consecutive doses and the minimum recommended interval in the post-loading period is 8 weeks [9].

### Switching patients to aflibercept 8 mg

Patients who do not respond to their current anti-VEGF treatment should have their diagnosis revisited. As with all agents, switching to aflibercept 8 mg can be considered in patients who have a suboptimal response (failure to achieve complete control of disease activity with a licensed drug dosing interval), in those patients where disease control is achieved but with an injection interval not tolerable or sustainable for that patient and when treatment harmonisation is required [3, 12].

Patients with persistent active disease despite receiving their prior drug at the minimal licensed interval or who are receiving treatment at intervals <q8 can be considered for switching to aflibercept 8 mg. A loading phase of three doses 1 month apart is recommended, followed by gradual extension of the treatment interval by two weeks based on treatment response.

In patients with stable disease receiving treatment at intervals ≥q8 with an alternative anti-VEGF, transitioning to aflibercept 8 mg may be considered to allow for later extending to intervals longer than 8 weeks or to align bilateral treatment. In these patients, consider switching to a matching treatment interval, given the short-term additional patient burden and the logistical requirements associated with switching and reloading. When evaluating a switch from aflibercept 2 to 8 mg, differences in dose and licensed posology should also be considered (Table 1).

### Bilateral disease

Treating bilateral disease at the same visit could reduce the burden of frequent visits and is aligned with current expert recommendations for anti-VEGF treatment in general, with certain precautions (such as preferably using different drug batches) [3, 17, 18]. There are limited data on the safety of bilateral treatment with aflibercept 8 mg or with concomitant use of other anti-VEGF agents. Bilateral injections can, however, be considered and discussed with patients.

Patients consenting to aflibercept 8 mg injections in their primary eye and receiving another intravitreal agent in their second eye should normally be switched over to aflibercept 8 mg in that second eye so that both eyes are harmonised, receiving the same therapy. Consideration needs to be given as to whether an 8-week minimum treatment interval is the best option for both eyes in that scenario.

Aligning treatment intervals should be discussed with the patient to minimise treatment burden and optimise service provision. For small interval differences, using the lowest common denominator is recommended, such as both eyes every 10 weeks if one eye requires 10-week and the other 12-week intervals. For larger interval differences, visits can be scheduled so the eye on the longer treatment interval is treated at every second appointment.

### Switching patients from aflibercept 8 mg

In patients who show signs of disease activity while receiving treatment at q8 intervals, it is advisable to continue at this interval for at least another dose before considering switching to another agent, provided VA remains stable and there is a consistent trend toward fluid resolution. In the PULSAR trial, only two patients withdrew from the trial owing to lack of efficacy by Week 48, both in the aflibercept 2 mg group [7]. Also, data suggest that residual low-volume subretinal fluid may not be detrimental to visual outcomes over time, but further studies are needed to inform clinical practice [24, 25]. The management of patients requiring 8-weekly dosing with residual fluid is, therefore, guided by clinician discretion following a discussion with the patient. Caution should be taken to ensure any visual loss is due to disease activity before changing the treatment interval or switching to a different agent.

It is inadvisable to switch to first-generation anti-VEGF agents (including aflibercept 2 mg, bevacizumab gamma and ranibizumab) in patients who display a suboptimal response to aflibercept 8 mg. However, this may also be considered in cases where the patient responded well to the previous anti-VEGF agent [12] but a switch to aflibercept 8 mg is required to harmonise therapies in both eyes or reduce treatment burden.

### Discontinuing

It is important to discuss with patients that recurrence rates after discontinuation of anti-VEGF treatment are approximately one-third in the subsequent 1–2 years and any visual loss may not be regained [26, 27]. Therefore, it is advisable that treatment should be continued indefinitely even when patients reach a 24-week treatment interval with no signs of disease activity. However, discontinuation of treatment and regular follow-up may be considered after informed discussions between clinician and patient. When there is a diagnosis of late AMD (wet inactive) disease and/or there is no prospect of visual improvement, it may also be appropriate to discontinue treatment [3].

### Consenting

Best practice involves obtaining patient consent when switching therapeutic agents, when there is a significant change in posology of the current agent, or if there is a change to the clinical condition and/or the perceived benefit/risk to the patient [3]. The distinct posology, formulation and injection volume of aflibercept 8 mg compared with aflibercept 2 mg (Table 1), combined with its enhanced durability and superior drying effect potentially resulting in fewer injections over the same period, should be regarded as a material change in treatment requiring a renewed consent. Current evidence suggests that aflibercept 8 mg has a similar safety profile to the lower volume of aflibercept 2 mg; however, it may be beneficial to discuss with eligible glaucoma patients the potential risk of an intraocular pressure spike following intravitreal injection and the potential worsening of optic disc cupping associated with larger injection volumes in high-risk patients. Patients should be consented in accordance with national legislation, professional standards of practice and local institutional policies. Regardless of whether written or verbal consent is obtained, it is recommended that decision-making is a dynamic, ongoing process and discussions are documented. These discussions should include the risks and benefits of alternative approved second-generation treatments, such as faricimab [28].

### Monitoring – special considerations

#### OCT

Where capacity allows, each eye should undergo OCT monitoring at every visit unless the fellow eye has end-stage disease and any treatment would be futile [29]. This is to identify and treat new nAMD in the second eye early, which is associated with better long-term outcomes [30].

At present, it is not mandated that patients on treatment intervals longer than 16 weeks as part of a T&E posology undergo additional monitoring in the affected eye. However, long treatment intervals or discontinuation of treatment should not affect standard care monitoring of the other eye, which may require more frequent monitoring [6].

#### Intraocular pressure

Pre-injection intraocular pressure should be documented at least at baseline and at 12 months, or more frequently in high-risk patients (including those with glaucoma and/or advanced cupping). Clinicians should refer to local and national guidelines for measuring and managing post-injection intraocular pressure in high-risk patients, as well as in those experiencing pain or reduced VA immediately after an injection [20].

#### Fundus

Fundus examination or colour photography is warranted when VA declines or patients report a decline in visual function, even when OCT appearances are stable [29] to assist in diagnosing new haemorrhages in particular. Investigation with fundus fluorescein angiography and indocyanine green angiography should also be considered when there is a suboptimal response to treatment, diagnostic uncertainty or unexplained reduction in vision [29].

### Implications for implementation

Since our roundtable meeting, NHS England has published commissioning guidelines and a unified pathway for the management of nAMD with the aim of addressing service challenges and inconsistent treatment monitoring across NHS Trusts to ensure patients receive the best value treatment at the right time [12]. Our pathway, focused on being practical, is broadly aligned with the NHS England recommendations when there is a switch to aflibercept 8 mg and operationalizing it is dependent on local policies, capacity and service constraints.

### Future considerations

While the evidence of the real-world effectiveness of aflibercept 8 mg was limited at the time of our initial meeting, several studies have since been published. Overall, notable improvements in VA and/or anatomical outcomes with aflibercept 8 mg have been observed in both treatment-naïve and previously treated nAMD patients (including those previously treated with aflibercept 2 mg), along with the achievement of longer treatment intervals [31–35]. However, these benefits appear less pronounced in patients switching to aflibercept 8 mg because of suboptimal response while on other second-generation anti-VEGF agents, such as faricimab [36]. The safety profile of aflibercept 8 mg has also been corroborated by additional research. A few cases of retinal vasculitis have been identified but managed successfully [37, 38] and transient, mild increases in intraocular pressure have been observed, similar to other agents, which did not require urgent intervention [23, 39, 40].

However, some questions remain. It is unclear whether reloading switch patients leads to improved outcomes or facilitates longer future treatment intervals and which patients may benefit equally from a longer interval extension compared to a more cautious approach. Long-term, real-world studies are also needed to provide further insights into the durability and safety of aflibercept 8 mg. Findings from the phase IIIb ELARA (NCT06491914) and the ongoing real-world SPECTRUM (NCT06075147, NCT06398080) studies may provide more information on the safety and efficacy of 4-weekly and long, 24-weekly aflibercept 8 mg injections, respectively. International registries such as the FRB! Project (Fight Retinal Blindness) are expected to provide additional real-world evidence.

## Closing comments

The drive to improve patient outcomes is often challenged by rising demand and overextended eye care services. To address this, providers are increasingly turning to the latest anti-VEGF treatments to reduce the treatment burden on both patients and clinics. Long-acting and durable therapies, such as aflibercept 8 mg, offer a potential solution to alleviate some of this strain [7, 41] and, by enabling clinics to operate within capacity, can improve patient outcomes through timely treatment while reducing clinic costs [5, 42].

In the PULSAR clinical trial, aflibercept 8 mg demonstrated a comparable efficacy and safety profile to aflibercept 2 mg, with treatment intervals extended up to 24 weeks [7, 8]. The presented pathway is based on our clinical experience as UK medical retina specialists. It aims to provide clarity and guidance on how we believe aflibercept 8 mg should be used in clinical practice for the treatment of nAMD to achieve good visual and anatomic outcomes while offering a low treatment burden. The RCOphth [3] and NHS England [43] emphasise the importance of shared decision-making between clinicians and patients to determine the most appropriate therapy for each individual patient. This pathway can, therefore, be used as an aid to inform both discussions on service improvement projects with relevant stakeholders and conversations with patients when discussing optimal patient-centred care.

## Summary

### What is known about this topic

- T&E treatment regimens for nAMD can alleviate some burden for patients and service providers by reducing the number of injections
- Intravitreal aflibercept 8 mg, licensed in the UK in January 2024, produces similar clinical outcomes and has a safety profile consistent with that of aflibercept 2 mg in patients with nAMD, with extended dosing intervals of up to 24 weeks

### What this study adds

- Expert-led recommendations and a clinical care pathway from UK retina specialists help to inform the management of patients with nAMD with extended dosing of aflibercept 8 mg

## Data Availability

All data analysed in this review are publicly available in the articles listed in the reference section. No new data were generated.
